# Efficacy of Dietary Supplements to Reduce Liver Fat

**DOI:** 10.3390/nu12082302

**Published:** 2020-07-31

**Authors:** Brittanie Kilchoer, Anina Vils, Beatrice Minder, Taulant Muka, Marija Glisic, Lia Bally

**Affiliations:** 1 Department of Diabetes, Endocrinology, Nutritional Medicine, and Metabolism, Inselspital, Bern University Hospital, University of Bern, 3010 Bern, Switzerland; brittanie.kilchoer@students.unibe.ch (B.K.); anina.vils@students.unibe.ch (A.V.); 2 Public Health & Primary Care Library, University Library of Bern, University of Bern, 3012 Bern, Switzerland; beatrice.minder@ispm.unibe.ch; 3 Institute of Social and Preventive Medicine, University of Bern, 2013 Bern, Switzerland; taulant.muka@ispm.unibe.ch (T.M.); marija.glisic@ispm.unibe.ch (M.G.); 4 Swiss Paraplegic Research, 6207 Nottwil, Switzerland

**Keywords:** liver fat, non/alcoholic fatty liver disease, dietary supplements, magnetic resonance imaging, magnetic resonance spectroscopy, computed tomography

## Abstract

Liver fat accumulation is an important pathophysiological feature of non-alcoholic fatty liver disease that may be modulated by dietary supplements (DS). A systematic search of the literature was conducted for randomized controlled trials (RCTs) pertaining to the effect of a DS on liver fat as assessed using quantitative tomographic imaging in human adults. Where feasible, data were pooled, and meta-analyses conducted using random-effect model. Quality assessment was done according the Cochrane Collaboration’s tool for assessing risk of bias. Twenty RCTs, involving 1171 overweight and obese adults, of which 36% were females, with or without comorbidities, were included. Only RCTs assessing omega-3 fatty acids (*n* = 4) and resveratrol (*n* = 4) qualified for meta-analysis. Results did neither favor omega-3 (effect size −1.17; weighted mean difference (WMD) (95% confidence interval (CI)) −3.62, 1.28; *p* < 0.001) nor resveratrol supplementation (0.18; 95% CI −1.08, 1.43; *p* = 0.27). The findings of the qualitatively summarized RCTs suggested that catechins (*n* = 1), *Lactobacillus reuteri* (*n* = 1), and carnitine (*n* = 1) may reduce liver fat. All other DS did not show any influence. The current evidence is scarce, of limited quality and does not support DS use to reduce liver fat. Further well-designed trials are warranted.

## 1. Introduction

Non-alcoholic fatty liver disease (NAFLD) is the most prevalent liver disease in the world and ranges from isolated steatosis to advanced stages with non-alcoholic steatohepatitis (NASH) and fibrosis, increasing the risk of cirrhosis and hepatocellular carcinoma [1]. The accumulation of liver fat (LF), that characterizes hepatic steatosis, is a key feature of the complex pathophysiology underlying NAFLD. Important factors are an increased influx of fatty acids from the diet and peripheral fat stores as well as upregulated de novo lipogenesis [2]. Hepatic steatosis is defined as LF of at least 5% of liver weight or 5% of hepatocytes containing lipid vacuoles. Whilst liver biopsy is considered as the gold standard method to quantify LF content [3,4], its invasiveness limits its applicability. Imaging modalities such as computed tomography (CT), magnetic resonance imaging-proton density fat fraction (MRI-PDFF), and magnetic resonance spectroscopy (MRS) are considered acceptable alternatives to quantify LF.

Lifestyle modifications, including energy restriction and physical activity, represent the cornerstone for the prevention and treatment of LF accumulation by inducing weight loss [5], improving insulin sensitivity and reducing inflammation [2,6,7,8]. However, the use of dietary supplements (DS) may further contribute to improve hepatic lipid homeostasis.

DS are a concentrated source of nutrients and other bioactive compounds that are intended to supplement the diet [9] comprising specific macro- and micronutrients, phytochemicals, dietary fibers, as well as probiotics and medicinal mushrooms [10,11]. Several studies have explored the efficacy of different DS on LF in humans in various populations (e.g., children and adults), as stand-alone or combined interventions (e.g., physical exercise [12] and drugs [13]). Despite the great interest in this area, no systematic effort on summarizing and quantifying the evidence on this topic has been undertaken.

Therefore, the objective of this study was to summarize the available evidence and evaluate the efficacy of various DS on LF in randomized clinical trials with adults using CT, MRI-PDFF, or MRS.

## 2. Materials and Methods

### 2.1. Data Sources and Search Strategy

This review followed the guidelines of a recently published paper on how to perform a systematic review and meta-analysis [14], and is reported in accordance with the Preferred Reporting Items for Systematic Reviews and Meta-Analyses (PRISMA) statement. By using terms related to DS and liver fat, an information specialist (B.M.) searched five bibliographic databases (Embase.com, Medline Ovid, PubMed, Cochrane CENTRAL, Web of Science, and Google Scholar) before 6 May 2020 (Supplementary Materials Table S1).

### 2.2. Study Selection and Eligibility Criteria

Studies were included if they met the following inclusion criteria: (i) were randomized controlled trials (RCTs); (ii) were performed on outpatient adults of ≥ 18 years; (iii) included an intervention that comprised of DS assessing its independent effect (e.g., combined interventions such as DS and physical activity were not considered eligible) and a control arm; (iv) reported a change from baseline to end of study in LF measured by tomographic imaging (MRI-PDFF, MRS, or CT); and (v) the interval between the assessment was ≥ 24 h (in order to avoid missing any potential short-term effects of DS [15,16]). Two independent reviewers (B.K. and A.V.) screened the titles and abstract in the first round, and the full text in a second round.

### 2.3. Data Extraction

The two reviewers independently performed data extraction using a standardized protocol (PROSPERO number CRD42020153556) and predesigned form including relevant information on lead author, study location, study design, study participants characteristics (health status, age, body mass index), DS (intervention characteristics and duration), control characteristics, and LF assessment. In case of incomplete data, corresponding authors were contacted via email.

### 2.4. Assessing the Risk of Bias

The two reviewers independently evaluated risk of bias within each individual RCT using the Cochrane Collaboration’s tool [17]. A detailed assessment of the risk of bias can be found in the Supplementary Materials Table S2.

### 2.5. Data Synthesis and Analysis

The effect of the DS was defined as the difference between DS-induced vs. control-induced change in LF. In order to standardize the numerical values from all RCTs, the method by Hozo et al. [18] was used to calculate mean and standard deviations, when feasible. Random-effect models were used to obtain estimates of weighted mean differences and 95% confidence intervals. As sensitivity analysis, we showed results of fixed effect models in the forest plots. Heterogeneity was assessed using the Cochrane *χ*^2^ statistic and the *I*^2^ statistic and was determined as low (*I^2^* ≤ 25%), moderate (25% < *I^2^* < 75%), or high (*I^2^* ≥ 75%) [19]. All statistical analyses were conducted with STATA, Release 14 (Stata Corp, College Station, TX, USA). The trials that could not be quantitatively pooled were summarized qualitatively (*n* = 12).

## 3. Results

**Figure 1:**
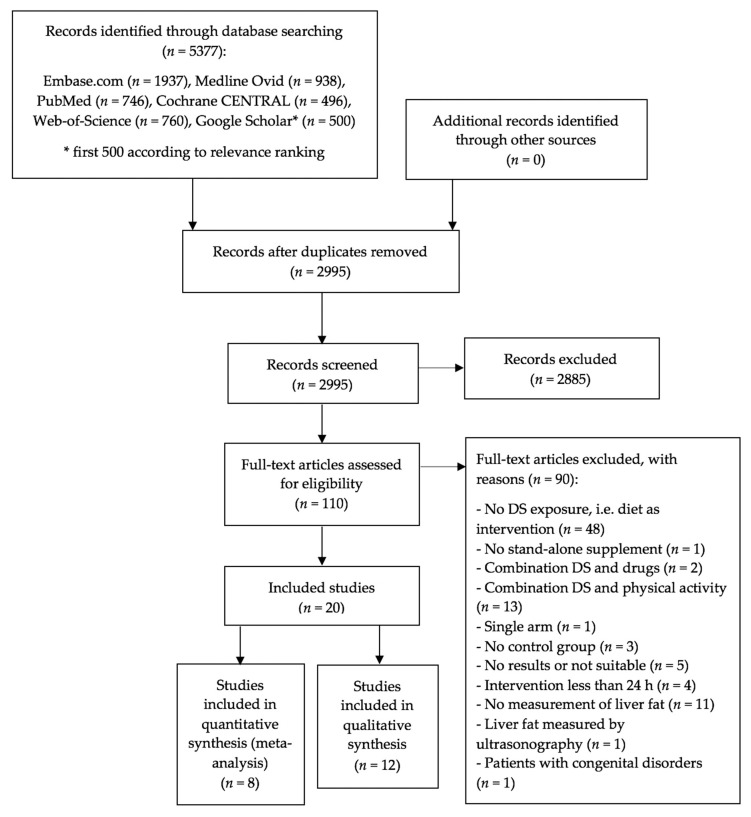
PRISMA flowchart showing the process for the inclusion of studies. Abbreviations: DS = dietary supplements, *n* = number of randomized controlled trials, PRISMA = Preferred Reporting Items for Systematic Reviews and Meta-Analyses.

The bibliographic searches identified 2995 unique abstracts (Figure 1).

After screening for titles and abstracts, 110 articles were considered for full-text assessment, of which 20 RCTs were eligible for the final analysis. Among those, eight trials contributed to the meta-analysis and 12 were included in the qualitative review.

### 3.1. Characteristics of RCTs

A summary of the most important characteristics of the 20 RCTs, based on 1171 patients of which 36% were female, can be found in Table 1. More detailed characteristics of each study are found in Supplementary Materials Table S3.

Eleven RCTs were performed in Europe, five in Asia, two in Australia, and one each in North and South America. No RCT studied normal-weight volunteers and all were performed in overweight and obese patients: one RCT included women with polycystic ovary syndrome (PCOS) and two RCTs studied non-alcoholic steatohepatitis (NASH) patients. Of the 11 RCTs studying NAFLD patients, two of them studied patients with type 2 diabetes mellitus (T2DM) and three with prediabetes or insulin resistance. Two RCTs studied exclusively overweight men without any other comorbidities, one RCT studied obese patients deficient in vitamin D (VD) and another RCT with prediabetes. Concerning the intervention, the DS were divided into the following categories: *n*-3 polyunsaturated fatty acids (*n* = 4; omega-3), phytochemicals (*n* = 8; 4 resveratrol, 1 pinitol, 1 catechins, and 2 resistant starches), probiotics and medicinal mushrooms (*n* = 4; 1 *Lactobacillus reuteri*, 1 *Lactobacillus* spp. plus *Bifidobacterium bifidum*, 1 *Bifidobacterium animalis*, and 1 *Cordyceps militaris*), vitamins (*n* = 3; 2 vitamin D, 1 nicotinamide riboside), and amino acid derivatives (*n* = 1; carnitine). The comparator entailed olive oil (*n* = 3), micro-cellulose (*n* = 1), amylopectin (*n* = 2), maltodextrin (*n* = 1), and inert substances, i.e., placebo (*n* = 11). Two RCTs only used lifestyle recommendation as a comparator. The duration of the interventions ranged between 4–78 weeks. Regarding the different methods of LF quantification, 12 RCTs used MRS, five RCTs used MRI-PDFF, and three RCTs used CT.

**Figure 2:**
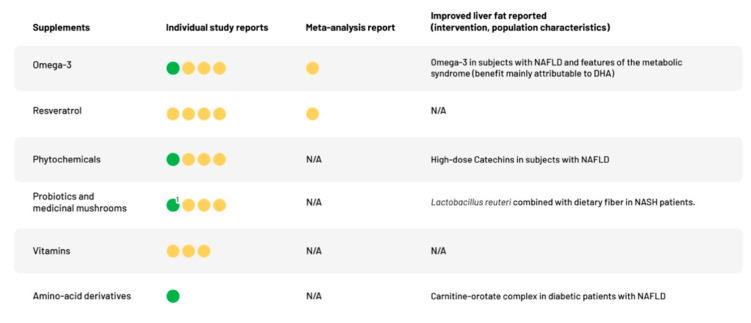
Summary of the most important findings. Each circle represents a study included in the analysis. Green circles indicate positive (reduction in liver fat compared to control) findings whereas orange circles denote negative findings (no significant difference between intervention and control). Abbreviations: DHA = docosapentanoic acid, NALFD = non-alcoholic fatty liver disease, NASH = non-alcoholic steatohepatitis, N/A = non-available. ^1^ Statistically significant reduction in liver fat content was reported in the intervention group. No change in liver fat was observed in the control group (*p*-value for the between group difference was not reported; detailed results in Supplementary Materials Table S4).

Figure 2 graphically illustrates key findings of the analysis. A descriptive summary of the qualitatively assessed RCTs are provided in Table 2 and a more detailed numerical analysis in Supplementary Materials Table S4.

#### 3.1.1. Omega-3

**Figure 3:**
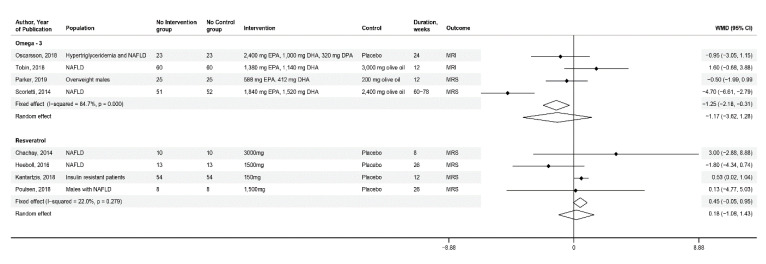
Meta-analysis of the effect of omega-3 and resveratrol supplementation on liver fat using a random-effects model. Abbreviations: CI = confidence interval, DHA = docosahexaenoic acid, DPA = docosapentaenoic acid, EPA = eicosapentaenoic acid, MRI = magnetic resonance imaging, MRS = magnetic resonance spectroscopy, NAFLD = non-alcoholic fatty liver disease, WMD = weighted mean difference.

We identified four RCTs [20,21,22,23] investigating the efficacy of omega-3 (combination of eicosapentaenoic acid (EPA) and docosahexaenoic acid (DHA)) using MRS (*n* = 2) or MRI-PDFF (*n* = 2) in a total of 375 patients. Scorletti et al. [20] examined the effect of daily dose of 3360 mg of omega-3 vs. 2400 mg of olive oil over 60 to 78 weeks using MRS in 103 subjects with NAFLD and features of the metabolic syndrome. Parker et al. [21] studied 50 overweight males without comorbidities for 12 weeks with 2000 mg of omega-3 vs. 2000 mg of olive oil using MRS. Oscarsson et al. [22] studied the effects of daily 4000 mg of omega-3 vs. placebo using MRI-PDFF in 46 overweight adults NAFLD and hypertriglyceridemia over 12 weeks. Tobin et al. [23] included 176 adults with NAFLD and measured the effects of a daily dosage of 2520 mg of omega-3 vs. 3000 mg of olive oil using MRI-PDFF. When pooling together the findings from those four RCTs, the calculated weighted mean difference (WMD) was −1.17 with a 95% confidence interval (CI) of −3.62 to 1.28. Heterogeneity was high with an I^2^ = 84.7%, *p* < 0.001 (Figure 3).

Due to differences in LF assessment among the four RCTs, we performed a separate analysis pooling together RCTs which assessed the LF content using MRI-PDFF and MRS respectively and results remained in line with the overall observation (Supplementary Materials Figure S1).

#### 3.1.2. Phytochemicals

Out of the eight RCTs [24,25,26,27,28,29,30,31] assessing phytochemicals, four RCTs qualified for qualitative analysis only and used the DS pinitol [28], catechins [29], and resistant starches [30,31]. The other four RCTs [24,25,26,27], assessing the efficacy of resveratrol using MRS in a total of 174 patients, were included in a meta-analysis. Chachay et al. [24], Heebøll et al. [25], and Poulsen et al. [27] studied patients with NAFLD. Kantartzis et al. [26] studied insulin resistant patients. The daily doses of resveratrol supplementation were 3000 mg for 8 weeks vs. micro-cellulose [24], 1500 mg for 26 weeks vs. placebo [25,27] or 150 mg for 12 weeks vs. placebo [26]. None of the RCTs demonstrated a significant change in LF content and this was in line with the pooled estimated of our meta-analysis (WMD 0.18; 95% CI −1.08, 1.43). The heterogeneity was low with an I^2^ of 22%, *p* = 0.27 (Figure 3).

Among the four RCTs included in qualitative synthesis, besides an indication that low doses of pinitol and catechins may improve the LF, other phytochemicals did not show beneficial effects on LF content.

In particular, Lee et al. [28] assessed the effects of a 12 week regimen of 300 mg (low) or 500 mg (high) of daily pinitol vs. placebo in 90 subjects with NAFLD using MRI-PDFF. Despite a lack of significant differences among the treatment groups, LF content decreased significantly in the low-dose group at 12 weeks compared to baseline (*p* = 0.01), whereas no significant change was detected with the higher pinitol dose (*p*-value not reported). Of note, the baseline of LF in the low-dose pinitol group (20.0 ± 1.5%) was higher than in the high-dose group (14.8 ± 1.9%). Therefore, the potential of the DS to decrease LF in the low-dose group was greater than in the high high-dose group, which could be an explanation for the greater decrease in LF in the low-dose group. Such dependence from baseline LF content was notably also observed with other DS. For instance, Cussons et al. reported that LF decreased with omega-3 only when individuals started with a higher LF amount (LF percentage >5%) [32].

Sakata et al. [29] contrasted 200 mg (low) or 1080 mg (high) of catechins, a green tea component, with placebo in 17 NAFLD patients for 12 weeks using CT. The high-dose catechin group significantly reduced LF as evidenced by an increase in the liver–spleen attenuation, whereas no change in LF content was observed in the low-dose catechin and placebo group (between group difference high dose vs. placebo: 14.6% (6.5%), *p* < 0.05).

In addition, two RCTs [30,31] explored the use of resistant starches (RS) on LF in overweight/obese adults using MRS. Johnston et al. [30] contrasted a daily intake of Hi-Maize^®^ 260 [33] with Amioca^®^ in 20 insulin-resistant but normoglycemic adults for 12 weeks and did not find any significant effect between both groups: 1.4% (4.6%), *p* = not significant (n.s.).

Peterson et al. [31] contrasted the same DS with Amioca^®^ over 12 weeks in 68 overweight adults with prediabetes. Again, there was no statistically significant difference between groups: −1.34% (4.0%), *p* = 0.23.

#### 3.1.3. Probiotics and Medicinal Mushrooms

Four RCTs investigated stand-alone probiotics, probiotics combined with prebiotics (synbiotics), and medicinal mushrooms, of which two in NASH patients [34,35], one in NAFLD patients [36] and one in patients with elevated liver enzymes [37]. Ferolla et al. [34] compared the effects of *Lactobacillus reuteri* (0.1 × 10^9^ CFU/d) combined with dietary fiber (4000 mg) vs. lifestyle recommendations alone over 13 weeks in 50 NASH patients using MRI-PDFF. The intervention group showed a statistically significant reduction in LF content of −2.5% (4.5%), *p* = 0.03, whereas LF in the control remained unchanged (*p*-value for the between group difference not reported). Scorletti et al. [36] contrasted 1.0 × 10^9^ CFU/d of *Bifidobacterium animalis* (subspecies *lactis* BB-12), combined with fructo-oligosaccharides with maltodextrin 8000 mg/d in 104 NAFLD patients over 44–61 weeks using MRS. The effect of the synbiotic vs. placebo did not significantly differ (between group difference 2.3% (2.6%), *p* = 0.30). Wong et al. [35] studied 20 NASH patients and assessed the effects of the probiotic mixture *Lactobacillus plantarum, L. delbrueckii* spp. *bulgaricus, L. acidophilus, L. rhamnosus* and *Bifidobacterium bifidum* (0.2 × 10^9^ CFU/d over 26 weeks) vs. lifestyle recommendations alone using MRS. Although there was a significant LF reduction in the intervention group −7.7% (9.8%), *p* = 0.03, the difference was not statistically significant between both groups −6.8% (2.0%), *p* = 0.07. Heo et al. [37] examined the effects of the ascomycetes *Cordyceps militaris* (1500 mg/d) vs. placebo in 57 patients over 4 weeks using CT. In both the intervention and control group, the change in liver-to-spleen attenuation did not significantly differ from baseline: 21.4% (45.1%) vs. 9.6% (11.4%; both mean (standard error of the mean (SEM))), *p* = 0.10.

#### 3.1.4. Vitamins

Three RCTs [38,39,40] examined the effects of vitamin supplementation. Barchetta et al. [38] investigated the effects of 2000 IU/d of VD vs. placebo in 55 patients with NAFLD and T2DM over 24 weeks using MRI-PDFF. When compared with placebo, VD did not significantly reduce LF at the end of the trial (0.3% (1.3%), *p* = 0.57). Wamberg et al. [39] studied VD (7000 IU/d) vs. placebo over 26 weeks in 43 obese, VD-deficient subjects using MRS. Similarly, VD did not significantly reduce LF when compared with placebo (0.05% (0.04%), *p* = 0.51). Dollerup et al. [40] investigated the effects of 2000 mg daily NR, a member of the vitamin B-3 family, vs. placebo over 12 weeks in 40 obese men using MRS. LF difference between placebo and NR was not statistically significant (−1.8% (1.7%), *p* = 0.13).

#### 3.1.5. Amino-Acid Derivative

Only one RCT investigated the effects of a DS containing an amino acid derivative. Bae et al. [41] evaluated daily intake of 2472 mg of carnitine-orotate complex vs. placebo for 12 weeks in 72 diabetic patients with NAFLD using CT. LF was significantly reduced by carnitine when compared with placebo (−5.5% (5.4%), *p* = 0.01), as demonstrated by a significantly greater increase in liver-to-spleen attenuation.

### 3.2. Further Outcomes and Safety

Only one study reported a significant reduction in body weight in the intervention (*Lactobacillus reuteri*) [34] but not in the control group. This study further suggested a LF-reducing effect. A decrease in liver enzymes was shown in three RCTs [28,29,35] (pinitol, catechins, *Lactobacillus* spp.) that also demonstrated a significant decrease in LF. Of the two RCTs [24,41] (omega-3, carnitine) measuring an increase in liver enzymes, only the carnitine trial was associated with a decrease in LF. None of the two trials including NASH patients [34,35] reported a significant effect of DS on liver fibrosis. Of the 11 trials including NAFLD patients only three [20,29,41] showed a beneficial effect of DS on LF (carnitine, catechins, omega-3). Only the RCT by Heebøll et al. [25] assessing resveratrol reported a serious safety event with high likelihood of causality. In this case, febrile leukopenia and thrombocytopenia occurred after 10 days of resveratrol treatment and returned after repeated exposure.

### 3.3. Assessment of Bias and Heterogeneity

Summarizing the risk of bias assessment (Supplementary Materials Table S2), four of the RCTs [25,26,38,41] were rated as good quality RCTs, half (*n* = 10), were rated as fair quality [21,22,23,24,27,28,29,31,35,40], and the remaining six RCTs as poor quality [20,30,34,36,37,39]. The most prominent cause for a poor quality RCT was a lack of allocation concealment or a lack of explanation for how the allocation was made.

Out of the 20 RCTs included in this systematic review and meta-analysis, two (10%) RCTs [37,39] did not declare funding source, whereas the remaining 18 RCTs did. Thirteen (55%) RCTs declared receiving industry funding, of which seven included author-industry ties [21,22,23,26,36,40,41]. Three (15%) of RCTs [24,25,27] received funds from private non-industry sources and 11 (55%) RCTs [24,25,26,27,28,29,30,34,35,36,38] from public grants (Supplementary Materials Table S5).

## 4. Discussion

In this systematic review and meta-analysis, we summarized the available evidence from 20 RCTs investigating the effect of various DS on LF using quantitative tomographic imaging in adult populations. Identified RCTs were performed in overweight and obese people, with different degrees of impaired glucose homeostasis, mostly diagnosed with NAFLD using different criteria and showed a male predominance. Our findings do not support the intake of DS to reduce LF in these population.

Our results on omega-3 build upon a prior systematic review and meta-analysis by Parker et al. [42] assessing the effects of omega-3 on LF and liver function test. In contrast to our findings, Parker et al. concluded a positive effect of omega-3 on LF and liver function. Their approach differed from ours by including evidence from observational and intervention studies, stand-alone DS and combination of DS-diet intervention, and further considering biopsy or ultrasound-based LF assessment methods. Results from different LF measurements were pooled by Parker et al., whereas we stratified studies by quantification technique. Four new RCTs have been published since the analysis by Parker at al, which we have included in our meta-analysis.

Potential beneficial effects of omega-3 on LF include improvement of insulin sensitivity by decreasing hepatic tumor necrosis factor-α expression, decrease of fatty acid synthesis by negatively controlling sterol regulatory element binding protein-1c and increase of fatty acid oxidation by positively controlling peroxisome proliferator-activated receptor-α. Direct mechanistic links have been mainly shown in animal studies [43,44,45].

Similarly, positive effects of resveratrol on LF have been mainly proposed by animal studies and have been attributed to reduction in insulin resistance and improvements in lipid profile [46]. However, the results of our meta-analysis do not support a beneficial role of neither omega-3 nor resveratrol on LF in human populations.

The qualitative assessment of the 12 RCTs suggested that catechins, some probiotics, and carnitine may be effective in reducing LF. Catechins are known to reduce oxidative stress and the risk of T2DM in women [47] but its modulating effect on hepatic energy and lipid metabolism remain ill-defined [48]. Compared to the control condition, *Lactobacillus* [34] and *Bifidobacterium* spp. [35] showed a trend towards greater decrease in LF in NASH patients when compared to dietary advice alone. Underlying mechanisms of LF reduction by a change in the intestinal microbiota may involve the reduction of host fatty acid absorption and beneficial effects on energy homeostasis inducing weight loss [34,49,50]. A reduction in LF could however also be due to the prebiotic component included in the synbiotic, as the composite used does not allow to tease out the actual cause of LF reduction [34]. Positive effects of carnitine, an amino acid derivative transporting free fatty acid into mitochondria for beta-oxidation, have been linked with improved beta-oxidation, thereby diminishing lipid accumulation [41,51].

The qualitatively summarized eight RCTs which did not find a significant effect on steatosis studied vitamin D, nicotinamide riboside, resistant starch, *Lactobacillus* spp., *Bifidobacterium animalis*, and *Cordyceps militaris*. Benefits of vitamin D and nicotinamide riboside on LF were mainly suggested by experimental data showing an insulin-sensitizing effect [52,53]. Similarly, insulin resistance was shown to be improved by *Cordyceps militaris* in animal studies [54]. With respect to resistant starch, an insulin-sensitizing effect was also observed humans [55].

Although some DS were shown to reduce LF, the magnitude of the % fat reduction was consistently less than 5% which is inferior to the effects reported by lifestyle measures such as exercise and calorie restriction that achieve LF reductions of 25–50% over similar time periods [56,57]. This suggests that a negative energy balance is the driving force in reducing LF highlighting that well-designed lifestyle management programs still remains more effective in reducing liver fat than DS. Although preclinical studies have shown promising effects, most of these have not been translatable into clinical outcomes. Of note, lack of consistency between preclinical and clinical research further emphasizes the importance of good practices for DS studies in the preclinical setting, including optimized use of in vitro, in vivo and in silico models [58].

### 4.1. Strengths and Limitations of Current Systematic Review

To our knowledge, this is the first systematic review and meta-analysis that analyzes the role of DS on LF in RCTs using reliable quantitative imaging methods. This review was conducted in accordance with the Cochrane guidelines [17] and we used the Cochrane risk of bias tool [19] to rate the quality of the evidence. In order to reduce the risk of publication bias, a highly sensitive search strategy was created, and additional resources were searched including ClinicalTrials.gov. Furthermore, our research question is succinct and precise, focused only on liver fat changes, and only with the use of tomographic imaging.

Nevertheless, we acknowledge a number of limitations: The overall risk of bias in the included RCTs was relatively high. Limitations in the study design included incomplete description of study populations (particularly with respect to diagnosing NAFLD and impaired glucose homeostasis) and background diet and activity level during the intervention and control period. Additionally, standardization of feeding and activity condition prior to the measuring of LF, which is known to substantially affect measured values [59], was only specified in a minority of trials. With respect to the study interventions, there was a high level of heterogeneity between studies assessing the same DS, only allowing for two DS types to be meta-analyzed. This heterogeneity was caused by differences in DS regimens (dose and duration), design of comparators, study populations, and outcome assessment methods. In line with the sex-dependence of NAFLD prevalence, there was a male predominance in the study population which may limit generalizability of findings to females. The two meta-analyses that were feasible only included four RCTs and the number of patients in each RCT was relatively low, where all except for two RCTs [20,23] involved less than 100 participants. Therefore, the results should be interpreted with caution and were not amenable for subset analysis.

### 4.2. Implications for Clinical Practice and Future Research

Our findings may provide useful guidance for clinicians counselling patients with NAFLD or at risk for developing it. In agreement with our findings, the recently published 2019 European Society for Clinical Nutrition and Metabolism (ESPEN) guidelines on nutritional management of liver diseases state until further data regarding their efficacy are available, DS cannot be recommended to treat NAFLD [8].

Of note, the present work only assessed the effect of DS on fatty change which is not necessarily correlated with advancement of fibrosis, and important risk factors for cardiovascular disease and mortality [60,61]. Thus, an important implication for future trials may be to assess the effect of DS on both LF and fibrosis. Novel magnetic resonance protocols combining PDFF measurement and elastography may open new avenues in this respect.

Insights provided by this work propose the following further directions to improve the evidence of DS trials in the field: (i) adequate sample size and length of study intervention; (ii) homogeneous, well-characterized and gender-balanced study populations; (iii) selection of appropriate outcomes and measurement techniques.

Ongoing RCTs exploring DS on LF and other features of NAFLD support the interest in the topic (ClinicalTrials.gov identifiers: NCT03513523, NCT03914495, NCT02568605) and may soon expand available evidence with additional data.

## 5. Conclusions

In conclusion, the present work does not support the use of DS to reduce LF across various obese and overweight patients with different degrees of comorbidities. Future clinical trials assessing efficacy of DS to reduce LF should be based on high-quality preclinical research and adopt rigorous experimental designs using homogenous and well-characterized populations, comparison to placebo and reliable quantitative imaging methods.

## Figures and Tables

**Table 1:** Characteristics of randomized controlled trials by dietary supplements.

	Omega-3	Phytochemicals (Incl. Resveratrol)	Probiotics	Vitamins	Amino Acid Derivatives
**Number of RCTs**	4	8	4	3	1
**Location**					
Europe	3	4	1	3	-
Asia	-	2	2	-	1
Australia	1	1	-	-	-
North America	-	1	-	-	-
South America	-	-	1	-	-
**Demographics and anthropometrics**					
Total participants (*n*)	375	369	231	138	72
Age (yrs)	52 (10.9) ^1^	49 (9.9) ^1^	49.7 (11.6) ^1^	53 (8.3) ^1^	51 (9.2) ^1^
BMI (kg/m^2^)	31.6 (6.2) ^1^	31.6 (3.6) ^1^	32.5 (4.8) ^1,2^	33 (3.5) ^1^	27.5 (3.2) ^1^
% women	40	36	38	35	31
**Specific restricted study population ^3^**					
NAFLD	3	5	1	1 ^4^	1 ^4^
NASH	-	-	2	-	-
PCOS	1	-	-	-	-
Prediabetic	-	1	-	-	-
Insulin-resistant	-	2	-	-	-
T2DM	-	-	-	1 ^4^	1 ^4^
Others	1	-	1	2	-
**Duration** (**weeks**)	12–78	8–26	4–61	12–26	12
**Liver fat measured by**					
MRS	3	6	2	2	-
MRI	-	-	-	1	-
MRI-PDFF	2	1	1	-	-
CT	-	1	1	-	1

^1^ Results are reported in mean (standard deviation (SD)). ^2^ Only three studies included in the calculation. ^3^ This column only serves as a simple overview of the different populations. Obviously, many of the diseases overlap. More detailed information and how the diseases are defined can be found in Table S3 in the Supplement. ^4^ Study exclusively included a population with diagnosed NAFLD and T2DM. Abbreviations: BMI = body mass index, CT = computed tomography, MRI = magnetic resonance imagery, MRS = magnetic resonance spectroscopy, NAFLD = non-alcoholic fatty liver disease, *n* = number of participants, NASH = non-alcoholic steatohepatitis, PCOS = polycystic ovary syndrome, PDFF = proton density fat fraction, RCTs = randomized controlled trials, T2DM = type 2 diabetes mellitus, yrs = years.

**Table 2:** Descriptive summary of the 12 randomized controlled trials that were analyzed qualitatively, investigating the associations between dietary supplements and liver fat.

Lead Author, Publication Year	Treatment Characteristics			Main Findings ^1^
	Dietary Supplement	Control	Duration	
**Phytochemicals**				
Lee et al., 2019	Pinitol, 300 mg or 500 mg	Placebo	12 weeks	Although there were no significant differences among the treatment groups in the LF content changes using MRI-PDFF, LF content decreased significantly in the low-dose group (*p* = 0.01), whereas no significant change was detected with the higher pinitol dose.^1^
Sakata et al., 2013	Catechins, 200 mg or 1080 mg	Placebo	12 weeks	The improvement in liver-to-spleen CT attenuation was 11.3% (2.8%) in the high density and −6.1% (12.1%) in the low-density intervention groups vs. −3.3% (8.5%) in the placebo group. Compared to control group, the difference was −2.8% (7.4%) for the low-dose and 14.6% (6.5%), *p* < 0.05 for the high-dose intervention group.
Johnston et al., 2010	Hi-Maize 260, 40,000 mg	Amioca	12 weeks	LF fat reduction was −1.7% (7.3%) in the intervention vs. −0.3% (4.5%) in the control group. The between group difference was 1.4% (4.6%) (*p* = n.s.), measured by MRS.
Peterson et al., 2018	Hi-Maize 260, 45,000 mg	Amioca	12 weeks	MRS measurements could not show any significant decrease in LF neither in the intervention 0.2% (6.6%) nor in the control group 1.6% (6.7%) and between both groups −1.3% (4.0%), *p* = 0.23.
**Probiotics and medicinal mushrooms**				
Ferolla et al., 2016	Synbiotic *(Lactobaccillus reuteri)*, 0.1 × 10^9^ CFU	Usual diet	13 weeks	LF, measured by MRI-PDFF, decreased by −2.5% (4.5%) in the intervention (*p* = 0.03) and by 3.0% (5.5%) in the control group (*p* = 0.15). The between group difference was 5.5% (3.3%) (*p*-value not reported).
Scorletti et al., 2020	Synbiotic (*Bifidobacterium animalis* (subspecies *lactis* BB-12)), 1.0 × 10^9^ CFU	Maltodextrin, 8000 mg	44–61 weeks	LF, measured by MRS, changed by −3.8% (4.3%) in the intervention and by −6.1% (4.0%) in the control group. No significant between group difference was detected 2.3% (2.6%), *p* = 0.30.
Wong et al., 2013	Probiotics (*Lactobaccillus plantarum, L. delbrueckii* spp. *bulgaricus, L. acidophilus, L. rhamnosus; Bifidobacterium bifidum*), 0.2 × 10^9^ CFU	Usual diet	26 weeks	LF, measured by MRS, decreased significantly in the intervention −7.7% (9.8%), *p* = 0.03, and remained static in the control group at −0.9% (4.9%), *p* = 0.15. No significant change between the groups was detected (between group difference −6.8% (2.0%), *p* = 0.07).
Heo et al., 2015	Ascomycetes (*Cordyceps militaris*), 1500 mg	Placebo	4 weeks	In analysis of the liver CT scan the mean ratio of change of Hounsfield increased by an average of 21.4% (45.1%; mean (SEM)) in the intervention group and 9.6% (11.4%; mean (SEM)) in the control group (*p* = 0.10).
**Vitamins**				
Barchetta et al., 2016	Vitamin D, 2000 IU	Placebo	24 weeks	Changes in LF, measured by MRI, of −0.4% (2.1%) in the intervention and of −0.7% (1.5%) in the control group, did not differ significantly between both groups 0.3% (1.3%; *p* = 0.57).
Wamberg et al., 2011	Vitamin D, 7000 IU	Placebo	26 weeks	LF, assessed by MRS as the arbitrary lipid:water ratio, changed by 0.05% (0.07%), *p* = 0.11 in the intervention group and by 0.00% (0.06%), *p* = 0.61 in the control group. The difference between the groups of 0.05% (0.04%) was not significant (*p* = 0.51).
Dollerup et al., 2018	Nicotinamide riboside, 2000 mg	Placebo	12 weeks	A 2% (2.6%) reduction in LF in the intervention group was measured by MRS compared to a 0.2% (2.6%) reduction in the control group. No significant difference between groups was found −1.8% (1.7%), *p* = 0.13.
**Amino-acid derivatives**				
Bae et al., 2015	Carnitine-orotate, 2472 mg	Placebo	12 weeks	On the hepatic CT analysis, mean changes in liver-to-spleen attenuation ratio were 6.2% (9.0%), *p* < 0.01 in the intervention group and 0.7% (8.1%), *p* = 0.58 in the control group. These results showed a significant difference in LF reduction between both groups 5.5% (5.4%), *p* = 0.01.

If available, results are reported in mean (SD). In case of other values, they are mentioned directly in the text. ^1^ Authors reported results graphically only. The data of this article was extracted from the box-plot graphic with the program Plot Digitizer and can be found in Table S4 in the Supplement. CFU = colony forming units, CT = computed tomography, LF = liver fat, MRI = magnetic resonance imagery, MRS = magnetic resonance spectroscopy, n.s. = non-significant, PDFF = proton density fat fraction, SD = standard deviation, SEM = standard error of the mean.

## Supplementary Materials

The following are available online at https://www.mdpi.com/2072-6643/12/8/2302/s1, Figure S1: Separate omega-3 meta-analysis, Table S1: Detailed search strategy, Table S2: Risk of bias assessment of the randomized controlled trials based on the Cochrane collaboration’s Tool, Table S3: Detailed characteristics of randomized controlled trials, Table S4: Numerical results of the 12 randomized controlled trials included in the qualitative synthesis, Table S5: Summary of the funding sources of all randomized controlled trials.

## References

[1] Paul S., Davis A.M. (2018). Diagnosis and Management of Nonalcoholic Fatty Liver Disease. JAMA.

[2] Asrih M., Jornayvaz F.R. (2014). Diets and nonalcoholic fatty liver disease: The good and the bad. Clin. Nutr..

[3] Zhang Y.N., Fowler K.J., Hamilton G., Cui J.Y., Sy E.Z., Balanay M., Hooker J.C., Szeverenyi N., Sirlin C.B. (2018). Liver fat imaging-a clinical overview of ultrasound, CT, and MR imaging. Br. J. Radio.

[4] Stern C., Castera L. (2017). Non-invasive diagnosis of hepatic steatosis. Hepatol. Int..

[5] Koutoukidis D.A., Astbury N.M., Tudor K.E., Morris E., Henry J.A., Noreik M., Jebb S.A., Aveyard P. (2019). Association of Weight Loss Interventions With Changes in Biomarkers of Nonalcoholic Fatty Liver Disease: A Systematic Review and Meta-analysis. JAMA Intern. Med..

[6] Abenavoli L., Milic N., Peta V., Alfieri F., De Lorenzo A., Bellentani S. (2014). Alimentary regimen in non-alcoholic fatty liver disease: Mediterranean diet. World J. Gastroenterol. WJG.

[7] Suárez M., Boqué N., Del Bas J.M., Mayneris-Perxachs J., Arola L., Caimari A. (2017). Mediterranean diet and multi-ingredient-based interventions for the management of non-alcoholic fatty liver disease. Nutrients.

[8] Plauth M., Bernal W., Dasarathy S., Merli M., Plank L.D., Schütz T., Bischoff S.C. (2019). ESPEN guideline on clinical nutrition in liver disease. Clin. Nutr..

[9] Incze M. (2019). Vitamins and Nutritional Supplements: What Do I Need to Know?. JAMA Intern. Med..

[10] Food and Drug Administration FDA 101: Dietary Supplements. https://www.fda.gov/consumers/consumer-updates/fda-101-dietary-supplements.

[11] European Food Safety Authority Food Supplements. https://www.efsa.europa.eu/en/topics/topic/food-supplements.

[12] Hallsworth K., Thoma C., Moore S., Ploetz T., Anstee Q.M., Taylor R., Day C.P., Trenell M.I. (2015). Non-alcoholic fatty liver disease is associated with higher levels of objectively measured sedentary behaviour and lower levels of physical activity than matched healthy controls. Frontline Gastroenterol..

[13] Cui J., Philo L., Nguyen P., Hofflich H., Hernandez C., Bettencourt R., Richards L., Salotti J., Bhatt A., Hooker J. (2016). Sitagliptin vs. placebo for non-alcoholic fatty liver disease: A randomized controlled trial. J. Hepatol..

[14] Muka T., Glisic M., Milic J., Verhoog S., Bohlius J., Bramer W., Chowdhury R., Franco O.H. (2019). A 24-step guide on how to design, conduct, and successfully publish a systematic review and meta-analysis in medical research. Eur. J. Epidemiol..

[15] Browning J.D., Baxter J., Satapati S., Burgess S.C. (2012). The effect of short-term fasting on liver and skeletal muscle lipid, glucose, and energy metabolism in healthy women and men. J. Lipid Res..

[16] Dusilová T., Kovář J., Drobný M., Šedivý P., Dezortová M., Poledne R., Zemánková K., Hájek M. (2019). Different acute effects of fructose and glucose administration on hepatic fat content. Am. J. Clin. Nutr..

[17] Higgins J.P., Altman D.G., Gotzsche P.C., Juni P., Moher D., Oxman A.D., Savovic J., Schulz K.F., Weeks L., Sterne J.A. (2011). The Cochrane Collaboration’s tool for assessing risk of bias in randomised trials. BMJ.

[18] Hozo S.P., Djulbegovic B., Hozo I. (2005). Estimating the mean and variance from the median, range, and the size of a sample. BMC Med. Res. Methodol..

[19] Higgins J.P., Thompson S.G., Deeks J.J., Altman D.G. (2003). Measuring inconsistency in meta-analyses. BMJ.

[20] Scorletti E., Bhatia L., McCormick K.G., Clough G.F., Nash K., Hodson L., Moyses H.E., Calder P.C., Byrne C.D. (2014). Effects of purified eicosapentaenoic and docosahexaenoic acids in nonalcoholic fatty liver disease: Results from the WELCOME* study. Hepatology.

[21] Parker H.M., Cohn J.S., O’connor H.T., Garg M.L., Caterson I.D., George J., Johnson N.A. (2019). Effect of fish oil supplementation on hepatic and visceral fat in overweight men: A randomized controlled trial. Nutrients.

[22] Oscarsson J., Önnerhag K., Risérus U., Sundén M., Johansson L., Jansson P.A., Moris L., Nilsson P.M., Eriksson J.W., Lind L. (2018). Effects of free omega-3 carboxylic acids and fenofibrate on liver fat content in patients with hypertriglyceridemia and non-alcoholic fatty liver disease: A double-blind, randomized, placebo-controlled study. J. Clin. Lipidol..

[23] Tobin D., Brevik-Andersen M., Qin Y., Innes J.K., Calder P.C. (2018). Evaluation of a high concentrate omega-3 for correcting the omega-3 fatty acid nutritional deficiency in non-alcoholic fatty liver disease (CONDIN). Nutrients.

[24] Chachay V.S., Macdonald G.A., Martin J.H., Whitehead J.P., O’Moore-Sullivan T.M., Lee P., Franklin M., Klein K., Taylor P.J., Ferguson M. (2014). Resveratrol Does Not Benefit Patients With Nonalcoholic Fatty Liver Disease. Clin. Gastroenterol. Hepatol..

[25] Heebøll S., Kreuzfeldt M., Hamilton-Dutoit S., Poulsen M.K., Stødkilde-Jørgensen H., Møller H.J., Jessen N., Thorsen K., Hellberg Y.K., Pedersen S.B. (2016). Placebo-controlled, randomised clinical trial: High-dose resveratrol treatment for non-alcoholic fatty liver disease. Scand. J. Gastroenterol..

[26] Kantartzis K., Fritsche L., Bombrich M., Machann J., Schick F., Staiger H., Kunz I., Schoop R., Lehn-Stefan A., Heni M. (2018). Effects of resveratrol supplementation on liver fat content in overweight and insulin-resistant subjects: A randomized, double-blind, placebo-controlled clinical trial. DiabetesObes. Metab..

[27] Poulsen M.K., Nellemann B., Bibby B.M., Stødkilde-Jørgensen H., Pedersen S.B., Grønbæk H., Nielsen S. (2018). No effect of resveratrol on VLDL-TG kinetics and insulin sensitivity in obese men with nonalcoholic fatty liver disease. DiabetesObes. Metab..

[28] Lee E., Lim Y., Kwon S.W., Kwon O. (2019). Pinitol consumption improves liver health status by reducing oxidative stress and fatty acid accumulation in subjects with non-alcoholic fatty liver disease: A randomized, double-blind, placebo-controlled trial. J. Nutr. Biochem..

[29] Sakata R., Nakamura T., Torimura T., Ueno T., Sata M. (2013). Green tea with high-density catechins improves liver function and fat infiltration in non-alcoholic fatty liver disease (NAFLD) patients: A double-blind placebo-controlled study. Int. J. Mol. Med..

[30] Johnston K.L., Thomas E.L., Bell J.D., Frost G.S., Robertson M.D. (2010). Resistant starch improves insulin sensitivity in metabolic syndrome. Diabet. Med..

[31] Peterson C.M., Beyl R.A., Marlatt K.L., Martin C.K., Aryana K.J., Marco M.L., Martin R.J., Keenan M.J., Ravussin E. (2018). Effect of 12 wk of resistant starch supplementation on cardiometabolic risk factors in adults with prediabetes: A randomized controlled trial. Am. J. Clin. Nutr..

[32] Cussons A.J., Watts G.F., Mori T.A., Stuckey B.G.A. (2009). Omega-3 fatty acid supplementation decreases liver fat content in polycystic ovary syndrome: A randomized controlled trial employing proton magnetic resonance spectroscopy. J. Clin. Endocrinol. Metab..

[33] Ingredion HI-MAIZE^®^ 260 Resistant Starch. https://apac.ingredion.com/ingredients/emea/himaize-260-22000b01.html.

[34] Ferolla S.M., Couto C.A., Costa-Silva L., Armiliato G.N.A., Pereira C.A.S., Martins F.S., Ferrari M.L.A., Vilela E.G., Torres H.O.G., Cunha A.S. (2016). Beneficial effect of synbiotic supplementation on hepatic steatosis and anthropometric parameters, but not on gut permeability in a population with nonalcoholic steatohepatitis. Nutrients.

[35] Wong V.W., Won G.L., Chim A.M., Chu W.C., Yeung D.K., Li K.C., Chan H.L. (2013). Treatment of nonalcoholic steatohepatitis with probiotics. A proof-of-concept study. Ann. Hepatol..

[36] Scorletti E., Afolabi P.R., Miles E.A., Smith D.E., Almehmadi A., Alshathry A., Childs C.E., Del Fabbro S., Bilson J., Moyses H.E. (2020). Synbiotics Alter Fecal Microbiomes, But Not Liver Fat or Fibrosis, in a Randomized Trial of Patients With Nonalcoholic Fatty Liver Disease. Gastroenterology.

[37] Heo J.Y., Baik H.W., Kim H.J., Lee J.M., Kim H.W., Choi Y.S., Won J.H., Kim H.M., Park W.I., Kim C.Y. (2015). The Efficacy and Safety of Cordyceps militaris in Korean Adults Who Have Mild Liver Dysfunction. J. Clin. Nutr..

[38] Barchetta I., Del Ben M., Angelico F., Di Martino M., Fraioli A., La Torre G., Saulle R., Perri L., Morini S., Tiberti C. (2016). No effects of oral vitamin D supplementation on non-alcoholic fatty liver disease in patients with type 2 diabetes: A randomized, double-blind, placebo-controlled trial. BMC Med..

[39] Wamberg L., Kampmann U., Stødkilde-Jørgensen H., Rejnmark L., Pedersen S.B., Richelsen B. (2013). Effects of vitamin D supplementation on body fat accumulation, inflammation, and metabolic risk factors in obese adults with low vitamin D levels—Results from a randomized trial. Eur. J. Intern. Med..

[40] Dollerup O.L., Christensen B., Svart M., Schmidt M.S., Sulek K., Ringgaard S., Stødkilde-Jørgensen H., Møller N., Brenner C., Treebak J.T. (2018). A randomized placebo-controlled clinical trial of nicotinamide riboside in obese men: Safety, insulin-sensitivity, and lipid-mobilizing effects. Am. J. Clin. Nutr..

[41] Bae J.C., Lee W.Y., Yoon K.H., Park J.Y., Son H.S., Han K.A., Lee K.W., Woo J.T., Ju Y.C., Lee W.J. (2015). Improvement of Nonalcoholic Fatty Liver Disease With Carnitine-Orotate Complex in Type 2 Diabetes (CORONA): A Randomized Controlled Trial. Diabetes Care.

[42] Parker H.M., Johnson N.A., Burdon C.A., Cohn J.S., O’Connor H.T., George J. (2012). Omega-3 supplementation and non-alcoholic fatty liver disease: A systematic review and meta-analysis. J. Hepatol..

[43] Alwayn I.P., Gura K., Nosé V., Zausche B., Javid P., Garza J., Verbesey J., Voss S., Ollero M., Andersson C. (2005). Omega-3 fatty acid supplementation prevents hepatic steatosis in a murine model of nonalcoholic fatty liver disease. Pediatric Res..

[44] Alwayn I.P., Andersson C., Zauscher B., Gura K., Nosé V., Puder M. (2005). Omega-3 fatty acids improve hepatic steatosis in a murine model: Potential implications for the marginal steatotic liver donor. Transplantation.

[45] Levy J.R., Clore J.N., Stevens W. (2004). Dietary n-3 polyunsaturated fatty acids decrease hepatic triglycerides in Fischer 344 rats. Hepatology.

[46] Gómez-Zorita S., Fernández-Quintela A., Macarulla M., Aguirre L., Hijona E., Bujanda L., Milagro F., Martínez J., Portillo M. (2012). Resveratrol attenuates steatosis in obese Zucker rats by decreasing fatty acid availability and reducing oxidative stress. Br. J. Nutr..

[47] Glisic M., Kastrati N., Gonzalez-Jaramillo V., Bramer W.M., Ahmadizar F., Chowdhury R., Danser A.J., Roks A.J., Voortman T., Franco O.H. (2018). Associations between Phytoestrogens, Glucose Homeostasis, and Risk of Diabetes in Women: A Systematic Review and Meta-Analysis. Adv. Nutr..

[48] Sivakumar S., Palsamy P., Subramanian S.P. (2010). Impact of D-pinitol on the attenuation of proinflammatory cytokines, hyperglycemia-mediated oxidative stress and protection of kidney tissue ultrastructure in streptozotocin-induced diabetic rats. Chem.-Biol. Interact..

[49] Ley R.E., Turnbaugh P.J., Klein S., Gordon J.I. (2006). Microbial ecology: Human gut microbes associated with obesity. Nature.

[50] Turnbaugh P.J., Ley R.E., Mahowald M.A., Magrini V., Mardis E.R., Gordon J.I. (2006). An obesity-associated gut microbiome with increased capacity for energy harvest. Nature.

[51] Noland R.C., Koves T.R., Seiler S.E., Lum H., Lust R.M., Ilkayeva O., Stevens R.D., Hegardt F.G., Muoio D.M. (2009). Carnitine insufficiency caused by aging and overnutrition compromises mitochondrial performance and metabolic control. J. Biol. Chem..

[52] Cantó C., Houtkooper R.H., Pirinen E., Youn D.Y., Oosterveer M.H., Cen Y., Fernandez-Marcos P.J., Yamamoto H., Andreux P.A., Cettour-Rose P. (2012). The NAD(+) precursor nicotinamide riboside enhances oxidative metabolism and protects against high-fat diet-induced obesity. Cell Metab..

[53] Zhou Q.G., Hou F.F., Guo Z.J., Liang M., Wang G.B., Zhang X. (2008). 1,25-Dihydroxyvitamin D improved the free fatty-acid-induced insulin resistance in cultured C2C12 cells. Diabetes Metab. Res. Rev..

[54] Choi S.B., Park C.H., Choi M.K., Jun D.W., Park S. (2004). Improvement of insulin resistance and insulin secretion by water extracts of Cordyceps militaris, Phellinus linteus, and Paecilomyces tenuipes in 90% pancreatectomized rats. Biosci. Biotechnol. Biochem..

[55] Maki K.C., Pelkman C.L., Finocchiaro E.T., Kelley K.M., Lawless A.L., Schild A.L., Rains T.M. (2012). Resistant starch from high-amylose maize increases insulin sensitivity in overweight and obese men. J. Nutr..

[56] Cheng S., Ge J., Zhao C., Le S., Yang Y., Ke D., Wu N., Tan X., Zhang X., Du X. (2017). Effect of aerobic exercise and diet on liver fat in pre-diabetic patients with non-alcoholic-fatty-liver-disease: A randomized controlled trial. Sci. Rep..

[57] Haufe S., Engeli S., Kast P., Böhnke J., Utz W., Haas V., Hermsdorf M., Mähler A., Wiesner S., Birkenfeld A.L. (2011). Randomized comparison of reduced fat and reduced carbohydrate hypocaloric diets on intrahepatic fat in overweight and obese human subjects. Hepatology.

[58] Sorkin B.C., Kuszak A.J., Bloss G., Fukagawa N.K., Hoffman F.A., Jafari M., Barrett B., Brown P.N., Bushman F.D., Casper S.J. (2020). Improving natural product research translation: From source to clinical trial. FASEB J..

[59] Wu B., Han W., Li Z., Zhao Y., Ge M., Guo X., Wu X. (2016). Reproducibility of Intra- and Inter-scanner Measurements of Liver Fat Using Complex Confounder-corrected Chemical Shift Encoded MRI at 3.0 Tesla. Sci. Rep..

[60] Angulo P. (2007). Obesity and nonalcoholic fatty liver disease. Nutr. Rev..

[61] Ekstedt M., Hagström H., Nasr P., Fredrikson M., Stål P., Kechagias S., Hultcrantz R. (2015). Fibrosis stage is the strongest predictor for disease-specific mortality in NAFLD after up to 33 years of follow-up. Hepatology.

