# Supplementary material for: Efficacy of Dietary Supplements to Reduce Liver Fat
Source: Nutrients. 2020 Jul 31;12(8):2302. doi: 10.3390/nu12082302 (PMC7469018; doi:10.3390/nu12082302)
Supplement: Supplementary file 1 [file nutrients-12-02302-s001.zip › nutrients-862948-supplementary.docx]

Online supplementary materials

Efficacy of dietary supplements to reduce liver fat

**Figure S1. Separate omega-3 meta-analysis**

**Table S1. Detailed search strategy**

**Table S2. Risk of bias assessment of the randomized controlled trials based on the Cochrane Collaboration’s Tool**

**Table S3. Detailed characteristics of randomized controlled trials**

**Table S4. Numerical results of the 12 randomized controlled trials included in qualitative synthesis**

**Table S5. Summary of the funding sources of all randomized controlled trials**

| 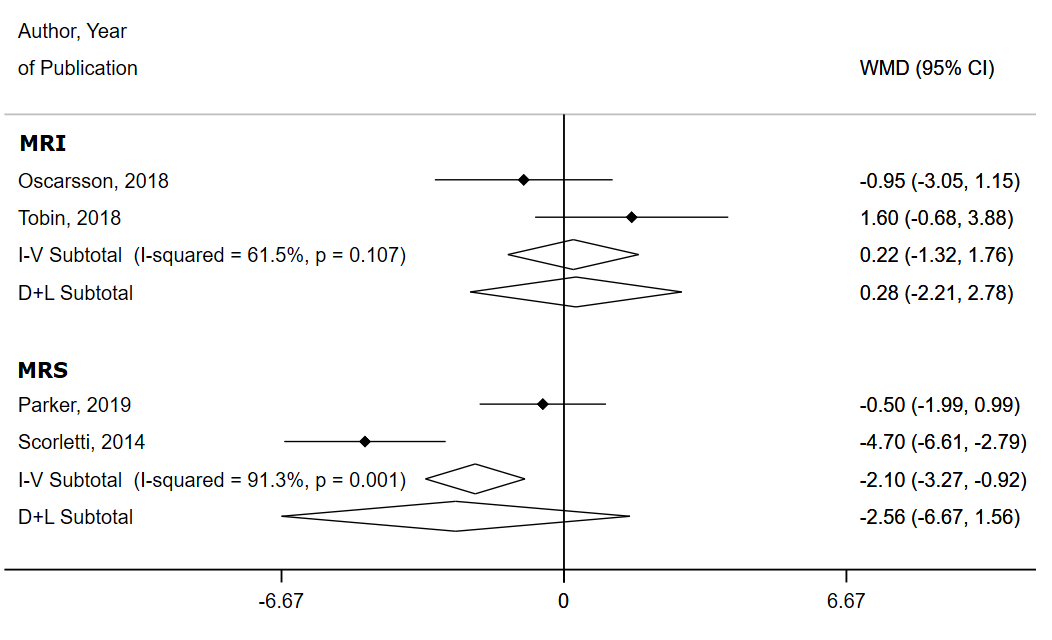 |
| --- |

**Figure S1.** Separation of the effects of omega-3 on liver fat measured by magnetic resonance imagery-proton density fat fraction or by magnetic resonance spectroscopy. Abbreviations: MRI = magnetic resonance imagery, MRS = magnetic resonance spectroscopy, WMD = weighted mean differences

**Table S1.** Detailed search strategy with keywords used in the various search engines.

| **Embase.com**  (1937 results) | ('nutrition'/exp OR 'diet'/exp OR 'diet therapy'/exp OR 'eating'/de OR 'food'/exp OR 'food intake'/exp OR 'nutrient'/exp OR 'caloric intake'/de OR 'caloric restriction'/de OR 'feeding behavior'/exp OR 'protein intake'/de OR 'carbohydrate diet'/exp OR 'dietary supplement'/de OR 'plant protein'/exp OR 'dietary fiber'/de OR 'fat intake'/exp OR (diet OR diets OR dietary OR dieti* OR nutrition* OR nutrient* OR macronutrient* OR macro-nutrient* OR micronutrient* OR micro-nutrient* OR food* OR ((dietary OR nutritional) NEAR/3 (fat* OR protein*)) OR carbohydrate* OR sugar* OR sodium OR calori* OR energy):ab,ti) AND ('fatty liver'/exp OR 'lipid liver level'/de OR (((intrahepat* OR hepatic OR liver) NEAR/3 (lipid* OR fat* OR triglyceride*)) OR IHL OR IHTG OR IHCL OR steatotic-liver* OR liver-steato* OR hepatic-steato* OR steatohepatitis OR hepatosteatos* OR fatty-liver* OR non-alcoholic-fatty-liver-disease* OR nonalcoholic-fatty-liver-disease* OR NAFLD* OR NASH):ab,ti) AND ('nuclear magnetic resonance scanner'/exp OR 'nuclear magnetic resonance spectroscopy'/de OR 'nuclear magnetic resonance imaging'/exp OR 'computer assisted tomography'/exp OR (magnetic-resonance OR MR-Spectroscop* OR MRS OR MRI OR computed-tomograph* OR CT):ab,ti) NOT ([animals]/lim NOT [humans]/lim) NOT ([Conference Abstract]/lim OR [Letter]/lim OR [Note]/lim OR [Editorial]/lim) |
| --- | --- |
| **Medline Ovid**  (938 results) | (exp "diet, food, and nutrition"/ OR exp Diet/ OR exp Diet Therapy/ OR exp Eating/ OR exp Food/ OR exp Nutrients/ OR exp Energy Intake/ OR exp Caloric Restriction/ OR exp Feeding Behavior/ OR exp Dietary Proteins/ OR exp Dietary Carbohydrates/ OR exp Dietary Supplements/ OR exp Dietary Fiber/ OR exp Dietary Fats/ OR (diet OR diets OR dietary OR dieti* OR nutrition* OR nutrient* OR macronutrient* OR macro-nutrient* OR micronutrient* OR micro-nutrient* OR food* OR ((dietary OR nutritional) ADJ3 (fat* OR protein*)) OR carbohydrate* OR sugar* OR sodium OR calori* OR energy).ab,ti.) AND (exp Fatty Liver/ OR (((intrahepat* OR hepatic OR liver) ADJ3 (lipid* OR fat* OR triglyceride*)) OR IHL OR IHTG OR IHCL OR steatotic-liver* OR liver-steato* OR hepatic-steato* OR steatohepatitis OR hepatosteatos* OR fatty-liver* OR non-alcoholic-fatty-liver-disease* OR nonalcoholic-fatty-liver-disease* OR NAFLD* OR NASH).ab,ti.) AND (exp Magnetic Resonance Spectroscopy/ OR exp Magnetic Resonance Imaging/ OR exp Tomography, X-Ray Computed/ OR (magnetic-resonance OR MR-Spectroscop* OR MRS OR MRI OR computed-tomograph* OR CT).ab,ti.) NOT (exp animals/ NOT humans/) NOT (letter* OR news OR comment* OR editorial* OR congresses OR abstracts).pt. |
| **PubMed**  **(746 results)**  *Focused on precision* | ((("Diet, Food, and Nutrition"[Mesh] OR "Diet"[Mesh] OR "Diet Therapy"[Mesh] OR "Eating"[Mesh] OR "Food"[Mesh] OR "Nutrients"[Mesh] OR "Energy Intake"[Mesh] OR "Caloric Restriction"[Mesh] OR "Feeding Behavior"[Mesh] OR "Dietary Proteins"[Mesh] OR "Dietary Carbohydrates"[Mesh] OR "Dietary Supplements"[Mesh] OR "Dietary Fiber"[Mesh] OR "Dietary Fats"[Mesh]) OR (diet[tiab] OR diets[tiab] OR dietary[tiab] OR dieting[tiab] OR dietitian*[tiab] OR nutrition*[tiab] OR nutrient*[tiab] OR macronutrient*[tiab] OR macro-nutrient*[tiab] OR micronutrient*[tiab] OR micro-nutrient*[tiab] OR food*[tiab] OR dietary fat*[tiab] OR nutritional fat*[tiab] OR dietary protein*[tiab] OR nutritional protein*[tiab] OR carbohydrate*[tiab] OR sugar*[tiab] OR sodium[tiab] OR calori*[tiab] OR energy[tiab])) AND (("Fatty Liver"[Mesh]) OR (intrahepat* lipid*[tiab] OR intrahepatic fat*[tiab] OR intrahepat* triglyceride*[tiab] OR hepatic lipid*[tiab] OR hepatic fat*[tiab] OR hepatic triglyceride*[tiab] OR liver lipid*[tiab] OR liver fat*[tiab] OR liver triglyceride*[tiab] OR IHL[tiab] OR IHTG[tiab] OR IHCL[tiab] OR steatotic liver*[tiab] OR liver steato*[tiab] OR hepatic steato*[tiab] OR steatohepatitis[tiab] OR hepatosteatos*[tiab] OR fatty liver*[tiab] OR non-alcoholic fatty liver disease*[tiab] OR nonalcoholic fatty liver disease*[tiab] OR NAFLD*[tiab] OR NASH[tiab])) AND (("Magnetic Resonance Spectroscopy"[Mesh] OR "Magnetic Resonance Imaging"[Mesh] OR "Tomography, X-Ray Computed"[Mesh]) OR (magnetic resonance[tiab] OR MRS[tiab] OR MRI[tiab] OR MRT[tiab] OR computed tomograph*[tiab] OR CT[tiab]))) NOT (Animals[MeSH Terms] NOT Humans[MeSH Terms]) NOT (letter[pt] OR news[pt] OR comment[pt] OR editorial[pt] OR congress[pt]) |

**Table S2.** Risk of bias assessment of the randomized controlled trials based on Cochrane risk of bias tool. The Cochrane Collaboration’s tool evaluates seven possible sources of bias: random sequence generation, allocation concealment, blinding of participants and personnel, blinding of outcome assessment, incomplete outcome data, selective reporting and other bias. Risk of bias of each item was judged as low (+), high (-) or unclear (?)..

| Lead author, year of publication | Random sequence generation | Allocation concealment | Selective reporting^1^ | Blinding of participants/personnel | Blinding of outcome assessment^2^ | Incomplete outcome | Other bias^3^ | Study  Quality^4^ |
| --- | --- | --- | --- | --- | --- | --- | --- | --- |
| Scorletti et al., 2014 | ? | ? | + | ? | ? | + | + | poor |
| Parker et al.,  2019 | + | + | + | + | + | + | - | fair |
| Oscarsson et al., 2008 | + | ? | + | ? | + | + | + | fair |
| Tobin et al.,  2018 | + | ? | + | + | ? | + | + | fair |
| Chachay et al.,  2014 | + | ? | + | + | + | + | + | fair |
| Heebøll et al.,  2016 | + | + | + | + | + | + | + | good |
| Kantartzis et al., 2018 | + | + | + | + | + | + | + | good |
| Poulsen et al., 2018 | ? | ? | + | + | + | + | + | fair |
| Lee et al.,  2019 | + | ? | + | + | + | + | + | fair |
| Sakata et al.,  2013 | ? | ? | + | ? | ? | + | + | fair |
| Johnston et al., 2010 | ? | ? | ? | ? | ? | ? | + | poor |
| Peterson et al., 2018 | + | ? | + | + | + | + | + | fair |
| Ferolla et al.,  2016 | ? | ? | ? | - | + | ? | + | poor |
| Scorletti et al.,  2020 | - | ? | + | + | ? | + | + | poor |
| Wong et al.,  2013 | + | + | + | ? | + | + | + | fair |
| Heo et al.,  2015 | + | ? | ? | ? | ? | ? | + | poor |
| Barchetta et al., 2016 | + | + | + | + | + | + | + | good |
| Wamberg et al., 2011 | + | ? | ? | + | + | - | + | poor |
| Dollerup et al., 2018 | + | ? | ? | + | + | + | + | fair |
| Bae et al.,  2015 | + | + | + | + | + | + | + | good |
| ^1^Trials clearly mentioning primary and secondary endpoints in the abstract, introduction or methods part and then comment every of them in the results part and discussion were considered to have a low risk of bias.  ^2^In case that blinding procedure was not sufficiently described we have considered studies to have low risk of bias in this domain as the outcome measurement was performed using a specific program and therefore there were not likely to be influenced by lack of blinding.  ^3^Because of the specific restricted study population (NAFLD, T2DM, BMI > 30.0 kg/m^2^ etc.) in every of our included RCT, we did not consider studies to have high risk of bias in this domain. We are aware that when comparing trials to each other, this may lead to heterogeneity.  ^4^Thresholds for Converting the Cochrane Risk of Bias Tool to AHRQ Standards (Good, Fair, and Poor)  Good quality: All criteria met (i.e. low for each domain)  Fair quality: One criterion not met (i.e. high risk of bias for one domain) or two criteria unclear, and the assessment that this was unlikely to have biased the outcome, and there is no known important limitation that could invalidate the results.  Poor quality: One criterion not met (i.e. high risk of bias for one domain) or two criteria unclear, and the assessment that this was likely to have biased the outcome, and there are important limitations that could invalidate the results. Poor quality: Two or more criteria listed as high or unclear risk of bias  *AHRQ = Agency for Healthcare Research and Quality, BMI = body mass index, NAFLD = non-alcoholic fatty liver disease, NASH = non-alcoholic steatohepatitis, RCT = randomized controlled trial, T2DM = type 2 diabetes mellitus* | | | | | | | | |

**Table S3.** Summary of all the randomized controlled trials included in this review.

| **Lead author, publication date** | **Location** | **N** | **Design** | **Patients** | | | | **Intervention** | **Control** | **Duration** | **Outcome assessment** |
| --- | --- | --- | --- | --- | --- | --- | --- | --- | --- | --- | --- |
|  |  |  |  | **Age (yrs)**  ***mean (SD)*** | **BMI**  **(kg/m^2^)**  ***mean (SD)*** | **%**  **Wo-men** | **Medical condition** |  |  |  |  |
| **Omega-3** | | | | | | | | | | | |
| Scorletti et al., 2014 | South-ampton, UK | 103 | Parallel  (1:1), double-blinded | 51 (10.3) | 33.1 (5.1) | 42 | NAFLD (*liver biopsy or imaging by US, MRI or CT)* | Omega-3 *(1840 mg EPA, 1520 mg DHA)*, 3360 mg | Olive oil, 2400 mg | 60 - 78 weeks | MRS  (%fat) |
| Parker et al.,  2019 | Sydney, Australia | 50 | Parallel  (1:1), double-blinded | 34 (10.6) | 27.9 (1.4) | 0 | Overweight males *(BMI 25.0–29.9 kg/m^2^, waist > 94 cm)*, no comorbidities | Omega-3 (*588 mg EPA, 412 mg DHA*), 2000 mg | Olive oil, 2000 mg | 12 weeks | MRS  (%fat) |
| Oscarsson et al., 2018 | Uppsala, Sweden | 46 | Parallel  (1:1:1)^1^, double-blinded | 60 (7.0) | 29.6 (13.0) | 41 | NAFLD *(MRI-PDFF > 5.5%)* | Omega-3 (*2000-2400 mg EPA, 600-1000 mg DHA, 40-320 mg DPA*), 4000 mg | Placebo | 12 weeks | MRI-PDFF (%fat) |
| Tobin et al.,  2018 | South-ampton, UK | 176 | Parallel  (1:1), double-blinded | 55 (12.1) | 32.4 (4.9) | 52 | NAFLD *(US)* | Omega-3 (*1380 mg EPA, 1140 mg DHA*),  2520 mg | Olive oil, 3000 mg | 24 weeks | MRI- PDFF (%fat) |
| **Phytochemicals** | | | | | | | | | | | |
| Chachay et al., 2014 | Brisbane, Australia | 20 | Parallel  (1:1), double-blinded | 48 (11.1) | 32.5 (2.7) | 0 | NAFLD *(US)* | Resveratrol, 3000 mg | Micro-cellulose | 8 weeks | MRS  (%fat) |
| Heebøll et al., 2016 | Aarhus, Denmark | 26 | Parallel  (1:1), double-blinded | 43 (11.2) | 32.1 (4.2) | --- | NAFLD *(liver biopsy)* | Resveratrol, 1500 mg | Placebo | 26 weeks | MRS  (%fat) |
| Kantartzis et al., 2018 | Tübingen, Germany | 112 | Parallel  (1:1), double-blinded | 48 (13.2) | 32.7 (4.0) | 52 | Insulin resistant *(HOMA-IR > 2.0)* and BMI ≥ 27.0 kg/m^2^ | Resveratrol, 150 mg | Placebo | 12 weeks | MRS  (%fat) |
| Poulsen et al., 2018 | Aarhus, Denmark | 16 | Parallel  (1:1), double-blinded | 49 (9.0) | 32.6 (3.1) | 0 | NAFLD *(MRS > 5.6%)* | Resveratrol, 1500 mg | Placebo | 26 weeks | MRS  (%fat) |
| Lee et al., 2019 | Seoul, Republic of Korea | 90 | Parallel  (1:1:1), double-blinded | 47 (2.0) | 27.6 (2.9) | 36 | NAFLD *(US)* | Pinitol, 300 mg or 500 mg | Placebo | 12 weeks | MRI-PDFF (%fat) |
| Sakata et al., 2013 | Kurume, Japan | 17 | Parallel  (1:1:1), double-blinded | 51 (13.1) | 29.2 (2.6) | 59 | NAFLD *(diagnostic criteria unclear)* | Catechins, 200 mg or 1080 mg | Placebo | 12 weeks | CT  (attenuation ratio) |
| Johnston et al., 2010 | London, UK | 20 | Parallel  (1:1), single-blinded | 48 (3.6) | 30.9 (1.4) | 40 | Insulin resistant (fasting insulin > 60 pM) | Hi-Maize 260 (RS type 2), 40,000 mg | Amioca (Amylopectin) | 12 weeks | MRS  (%fat) |
| Peterson et al., 2018 | Baton Rouge, Louisiana, USA | 68 | Parallel  (1:1), double-blinded | 55 (9.8) | 35.6 (4.7) | 66 | Overweight (*BMI > 27.0 kg/m^2^*) and prediabetes *(FPG 5.6-6.9 mM or HbA1c 5.7-6.4%)* | Hi-Maize 260 *(RS type 2)*, 45,000 mg | Amioca *(Amylopectin)* | 12 weeks | MRS  (%fat) |
| **Probiotics and medicinal mushrooms** | | | | | | | | | | | |
| Ferolla et al., 2016 | Minas Gerais, Brazil | 50 | Parallel  (1:1), not blinded | 57 (12.3) | 32.5 (3.9) | 76 | NASH *(liver biopsy)* | Synbiotic *(40,000 mg dietary fiber, Lactobaccillus reuteri),* 10,000 mg | Usual diet | 13 weeks | MRI-PDFF (%fat) |
| Scorletti et al.,  2020 | South-ampton, UK | 104 | Parallel  (1:1), double-blinded | 51 (12.6) | 33.1 (5.2) | 35 | NAFLD (*liver biopsy or imaging)* | Synbiotic (*8000 mg fructooligosaccharides, Bifidobacterium animalis (subspecies lactis BB-12*)) | Maltodextrin, 8000 mg | 44-61 weeks | MRS  (%fat) |
| Wong et al., 2013 | Hong Kong, China | 20 | Parallel  (1:1), not blinded | 49 (8.5) | 29.5 (5.1) | 35 | NASH *(liver biopsy)* | Probiotics (*Lactobaccillus plantarum, L. delbrueckii spp bulgaricus, L. acidophilus, L. rhamnosus; Bifidobacterium bifidum*), 20,000 mg | Usual diet | 26 weeks | MRS  (%fat) |
| Heo et al., 2015 | Seong-nam, Republic of Korea | 57 | Parallel  (1:1), double-blinded | 41 (10.1) | --- | 12 | ALT 1.5-3.0 x ULN, no systemic disease | Ascomycetes (*Cordyceps militaris*), 1500 mg | Placebo | 4 weeks  *(CT measurement at 8 weeks)* | CT  (% ratio of change of HU) |
| **Vitamins** | | | | | | | | | | | |
| Barchetta et al., 2016 | Rome, Italy | 55 | Parallel  (1:1), double-blinded | 59 (9.4) | 30.1 (4.4) | 35 | NAFLD *(confirmed by MRI)* and T2DM *(according to ADA criteria)* | Vitamin D, 2000 IU | Placebo | 24 weeks | MRI-PDFF  (%fat) |
| Wamberg et al., 2011 | Aarhus, Denmark | 43 | Parallel  (1:1), double-blinded | 40 (7.3) | 35.6 (3.2) | 71 | Obese (*BMI > 30.0 kg/m^2^*), VD deficient (25-OH VD < 50 nM) | Vitamin D, 7000 IU | Placebo | 26 weeks | MRS  (arbitrary units) |
| Dollerup et al., 2018 | Aarhus, Denmark | 40 | Parallel  1:1), double-blinded | 59 (7.9) | 32.9 (2.4) | 0 | Obese (*BMI > 30.0 kg/m^2^*), sedentary (*exercise < 30 min/d*), non-smokers | Nicotinamide riboside, 2000 mg | Placebo | 12 weeks | MRS  (%fat) |
| **Amino-acid derivatives** | | | | | | | | | | | |
| Bae et al., 2015 | Seoul, Republic of Korea | 72 | Parallel  (1:1), double-blinded | 51 (9.2) | 27.5 (3.2) | 31 | NAFLD *(ALT > 50-250 IU/L)* and T2DM *(HbA1c >6.4% or FPG > 7.2-16.7 mM)* | Carnitine-orotate, 2472 mg | Placebo | 12 weeks | CT  (attenuation ratio) |
| ^1^ third arm fenofibrate was not analyzed because of “DS and drug” was defined as exclusion criteria  AASLD = American Association for the Study of Liver Diseases, ADA = American Diabetes Association, C = control group, CT = computed tomography, DGB = Dietary Guide for Brazilians, DHA = docosahexaenoic acid, DPA = docosapentaenoic acid, DS = dietary supplement, DVFA = Danish Veterinary and Food Administration, EPA = eicosapentaenoic acid, FPG = fasting plasma glucose, HU = Hounsfield units, I = intervention group, MRI = magnetic resonance imagery, MRS = magnetic resonance spectroscopy, N = number of participants, NAFLD = non-alcoholic fatty liver disease, NASH = non-alcoholic steatohepatitis, PCOS = polycystic ovary syndrome, PDFF = proton density fat fraction, RS = resistant starches, SD = standard deviation, TG = triglycerides, T2DM = type 2 diabetes mellitus, ULN = upper limit of normal, US = ultrasonography, yrs = years | | | | | | | | | | | |

**Table S4.** Numerical results of the 12 randomized controlled trials included in the qualitative analysis.

| **Lead author, publication date** | **Type of intervention and daily dosage** | **Outcome assessment** | **Intervention group** | | | | **Control group** | | | | |
| --- | --- | --- | --- | --- | --- | --- | --- | --- | --- | --- | --- |
|  |  |  | ***N*** | **Baseline**  ***Mean***  ***(SD)*** | **End study**  ***Mean***  ***(SD)*** | **Difference**  ***Mean***  ***(SD/SEM*)*** | ***N*** | **Baseline**  ***Mean***  ***(SD)*** | **End study**  ***Mean***  ***(SD)*** | **Difference**  ***Mean***  ***(SD/SEM*)*** | |
| **Phytochemicals** | | | | | | | | | | | |
| Lee et al., 2019 | Pinitol,  300 mg | MRI-PDFF (%fat) | 30 | 19.3 (9.7)^1^ | 17.1 (11.3) | -2.2 (2.7) | 30 | 19.5 (9.3) | 17.2 (13.0) | -2.3 (2.9) | |
| Lee et al., 2019 | Pinitol,  500 mg | MRI-PDFF (%fat) | 30 | 13.1 (10.0)^2^ | 12.9 (12.0) | -0.2 (2.9) |  |  |  |  |  |
| Sakata et al., 2013 | Catechins,  200 mg | CT (attenuation ratio) | 5 | --- | --- | -6.1 (12.1) | 5 | --- | --- | -3.3 (8.5) | |
| Sakata et al., 2013 | Catechins,  1080 mg | CT (attenuation ratio) | 7 | 91.8 (4.6) | 101.8 (4.7) | 11.3 (2.8) |  |  |  |  |  |
| Johnston et al., 2010 | Hi-Maize 260,  40,000 mg | MRS (%fat) | 9 | 9.6 (10.0) | 9.3 (9.2) | -0.3 (4.5) | 9 | 8.8 (17.7) | 7.1 (13.0) | -1.7 (7.3) | |
| Peterson et al., 2018 | Hi-Maize 260,  45,000 mg | MRS (%fat) | 29 | 5.9 (7.7) | 6.1 (9.5) | 0.2 (6.5) | 25 | 6.9 (9.7) | 8.5 (12.4) | 1.62 (6.7) | |
| **Probiotics and medicinal mushrooms** | | | | | | | | | | | |
| Ferolla et al,. 2016 | Synbiotic *(*4000 mg dietary fiber, *Lactobaccillus reuteri),*  10,000 mg | MRI-PDFF  (%fat) | 27 | 15.1 (19.2) | 12.6 (13.6) | -2.5 (4.5) | 23 | 11.2 (15.4) | 14.2 (21.7) | 3.0 (5.5) | |
| Scorletti et al.,  2020 | Synbiotic (8000 mg fructooligo-saccharides, *Bifidobacterium animalis* (subspecies *lactis* BB-12)) | MRS (%fat) | 55 | 32.3 (24.8) | 28.5 (20.1) | -3.8 (4.3) | 49 | 31.3 (22.0) | 25.2 (17.2) | -6.1 (4.0) | |
| Wong et al., 2013 | Probiotics (*Lactobaccillus plantarum, L. delbrueckii spp bulgaricus, L. acidophilus, L. rhamnosus, Bifidobacterium bifidum*),  20,000 mg | MRS (%fat) | 10 | 22.6 (8.2) | 14.9 (7.0) | -7.7 (9.8) | 10 | 16.9 (6.1) | 16.0 (6.6) | -0.9 (4.9) | |
| Heo et al., 2015 | Ascomycetes (*Cordyceps militaris*),  1500 mg | CT (attenuation ratio) | 28 | --- | --- | 21.4 (45.1*) | 29 | --- | --- | 9.6 (11.4*) | |
| **Vitamins** | | | | | | | | | | |  |
| Barchetta et al., 2016 | Vitamin D,  2000 IU | MRI-PDFF (%fat) | 26 | 8.5 (8.5) | 8.1 (6.7) | -0.4 (2.1) | 29 | 6.8 (6.2) | 6.1 (5.1) | -0.7 (1.5) | |
| Wamberg et al., 2011 | Vitamin D,  7000 IU | MRS (arbitrary units) | 22 | 0.18 (0.16)^3^ | 0.23 (0.26) | 0.05 (0.07) | 21 | 0.18 (0.21) | 0.18 (0.18) | 0.00 (0.06) | |
| Dollerup et al., 2018 | Nicotinamide riboside,  2000 mg | MRS (%fat) | 20 | 11.3 (8.1) | 9.3 (8.1) | -2.0 (2.6) | 19 | 14.1 (8.1) | 13.9 (8.1) | -0.2 (2.6) | |
| **Amino acid derivative** | | | | | | | | | | |  |
| Bae et al., 2015 | Carnitine-orotate,  2472 mg | CT (attenuation ration) | 36 | -11.1 (10.3) | --- | 6.2 (9.0) | 36 | -8.8 (9.3) | --- | 0.7 (8.1) | |
| ^1^Authors reported results graphically only. The data of this article was extracted from the box-plot graphic with the program Plot Digitizer.  ^2^Liver fat content at baseline was significantly lower in the high-dose group than in the other groups.  ^3^MRI signal intensities (arbitrary units) are scaled by the MR system in an arbitrary fashion. These values are not comparable from study to study. Two significant digits were used to express these values.  CT = computed tomography, HU = Hounsfield units, MRI = magnetic resonance imagery, MRS = magnetic resonance spectroscopy, PDFF = proton density fat fraction, SD = standard deviation, SEM = standard error of the mean, --- = not reported | | | | | | | | | | | |

**Table S5.** Summary of the funding sources of all randomized controlled trials.

| **Lead author, publication date** | **Study Quality** | **Funding Sources** | | | | |
| --- | --- | --- | --- | --- | --- | --- |
|  |  | **Public** | **Private**  **(non-industry)** | **Industry** | **Author ties with industry** | **N/A** |
| Scorletti et al., 2014 | poor |  |  | x |  |  |
| Parker et al., 2019 | fair |  |  | x | x |  |
| Oscarsson et al., 2008 | fair |  |  | x | x |  |
| Tobin et al., 2018 | fair |  |  | x | x |  |
| Chachay et al., 2014 | fair | x | x |  |  |  |
| Heebøll et al., 2016 | good | x | x | x |  |  |
| Kantartzis et al., 2018 | good | x |  | x | x |  |
| Poulsen et al., 2018 | fair | x | x | x |  |  |
| Lee et al., 2019 | fair | x |  |  |  |  |
| Sakata et al., 2013 | fair | x |  | x |  |  |
| Johnston et al., 2010 | poor | x |  | x |  |  |
| Peterson et al., 2018 | fair |  |  | x |  |  |
| Ferolla et al., 2016 | poor | x |  |  |  |  |
| Scorletti et al., 2020 | poor | x |  | x | x |  |
| Wong et al., 2013 | fair | x |  |  |  |  |
| Heo et al., 2015 | poor |  |  |  |  | x |
| Barchetta et al., 2016 | good | x |  |  |  |  |
| Wamberg et al., 2011 | poor |  |  |  |  | x |
| Dollerup et al., 2018 | fair |  |  | x | x |  |
| Bae et al., 2015 | good |  |  | x | x |  |
| **Totals** | | **11** | **3** | **13** | **7** | **2** |

| s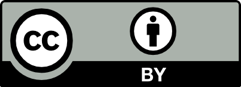 | © 2020 by the authors. Submitted for possible open access publication under the terms and conditions of the Creative Commons Attribution (CC BY) license (http://creativecommons.org/licenses/by/4.0/). |
| --- | --- |
